# Treatment with BKI-1748 after *Toxoplasma gondii* systemic dissemination in experimentally infected pregnant sheep improves fetal and lamb mortality and morbidity and prevents congenital infection

**DOI:** 10.1128/aac.01448-24

**Published:** 2024-12-31

**Authors:** Roberto Sánchez-Sánchez, Ana Huertas-López, Andrea Largo-de la Torre, Ignacio Ferre, Filippo Maria Dini, Michela Re, Javier Moreno-Gonzalo, Ryan Choi, Matthew A. Hulverson, Kayode K. Ojo, Samuel L. M. Arnold, Andrew Hemphill, Wesley C. Van Voorhis, Luis Miguel Ortega-Mora

**Affiliations:** 1 SALUVET, Animal Health Department, Faculty of Veterinary Sciences, Complutense University of Madrid, Ciudad Universitaria s/n 16734 , Madrid, Community of Madrid, Spain; 2 Animal Health Department, University of Murcia 571776 , Murcia, Region of Murcia, Spain; 3 SALUVET-Innova S.L., Faculty of Veterinary Sciences, Complutense University of Madrid, Ciudad Universitaria s/n 16734 , Madrid, Community of Madrid, Spain; 4 Department of Veterinary Medical Sciences, University of Bologna 9296 , Bologna, Emilia-Romagna, Italy; 5 Animal Medicine and Surgery Department, Faculty of Veterinary Sciences, Complutense University of Madrid, Ciudad Universitaria s/n 571788 , Madrid, Community of Madrid, Spain; 6 Center for Emerging and Re-emerging Infectious Diseases (CERID), Division of Allergy and Infectious Diseases, Department of Medicine, University of Washington 205280 , Seattle, Washington, USA; 7 Department of Pharmaceutics, University of Washington 312750 , Seattle, Washington, USA; 8 Institute of Parasitology, Vetsuisse Faculty, University of Berne, Berne, Switzerland; The Children's Hospital of Philadelphia, Philadelphia, Pennsylvania, USA

**Keywords:** *Toxoplasma gondii*, sheep, congenital toxoplasmosis, BKI-1748, treatment

## Abstract

Drug development for congenital toxoplasmosis is challenging since first-line therapy has a high rate of adverse effects and exhibits suboptimal efficacy. Bumped kinase inhibitors (BKIs), targeting protein kinases with small gatekeeper residues, have been found to be effective against *Toxoplasma gondii*. The efficacy of BKI-1748 administered later than 2 days post-infection (p.i.), a scenario that may better reflect its real-world use as a therapeutic candidate, has not been investigated in *T. gondii*-infected pregnant sheep. For this purpose, 19 pregnant sheep were assigned to three experimental groups. Group 1 (G1, *n* = 8) and group 2 (G2, *n* = 8) were dosed orally with 10 TgShSp1 sporulated oocysts at 90 days of gestation (dg). Animals from group 3 (G3, *n* = 3) were simultaneously mock dosed with phosphate-buffered solution (PBS). In G1, BKI-1748 was administered orally from day 7 p.i. (fever and increased serum IFNγ levels) onward, maintaining drug exposure for 20 days (10 doses at 15 mg/kg every 2 days). Treated animals (G1) exhibited significantly lower rectal temperatures (on days 8 and 9 p.i.), serum IFNγ levels (on day 10 p.i.), and specific IgG levels when compared with non-treated animals (G2). At delivery, significantly higher percentages of healthy lambs were found in infected/treated sheep in G1 (73.3%) and in uninfected sheep in G3 (80%) compared with infected/untreated sheep in G2 (31.3%). Concerning congenital transmission, parasite DNA was neither detected in placenta nor target tissues (brain and lungs) from the fetuses/lambs in G1(infected/treated) and G3 (uninfected). By contrast, parasite DNA was detected in all placentas and lambs from G2 (infected/untreated), except for one sheep that aborted on day 13 p.i.

## INTRODUCTION

Toxoplasmosis is a zoonotic disease caused by the apicomplexan parasite *Toxoplasma gondii,* affecting around one-third of the world population. Waterborne (by ingestion of oocysts) and foodborne (vegetables/fruits contaminated with oocysts or meat containing bradyzoites) are the main routes of *T. gondii* infection in humans (1). Congenital *T. gondii* infections in humans, with a global estimated annual incidence of 200,000 cases, are very relevant due to the clinical consequences for newborns (2). Most children with congenital toxoplasmosis are developmentally normal, but up to 4% die or have evidence of permanent neurological damage or bilateral visual impairment during the first years of life (3–5). Early gestational infection with *T. gondii* is clinically more severe for the fetus, although mother-to-child transmission is more efficient in the latter half of gestation (6). In small ruminants, postnatal infections are caused by oocyst contamination in food and water. *T. gondii* is responsible for 10%–23% of ovine abortions in Europe and the USA, and 3%–54% in the Middle East and South America (7), causing important economic losses in sheep flocks (8). In addition, consumption of raw or poorly cooked ovine meat is an important source of *T. gondii* infection in humans, at least in some countries (9).

For the treatment of congenital toxoplasmosis in humans, aiming to prevent newborn sequelae, prenatal screening is cost-effective as compared with neonatal screening (10), and the treatment is usually successful if applied within 3 weeks post-infection and commonly fails if applied after 8 weeks of infection (3, 4, 11–13). In the case of parasite detection in amniotic fluid immediately after 18 weeks of gestation, or in cases with high probability of fetal infection, a drug combination of sulfadiazine and pyrimethamine (folate inhibitors) is recommended (14–16). Some studies addressing the efficacy of this treatment reported a reduction in the severity of congenital toxoplasmosis; however, other studies yielded contradictory results. These discrepancies might be explained by the important limitations in these studies, such as the lack of an untreated control group or the uncertainty about the time lapse between maternal infection and onset of treatment, that possibly biased the results (4, 12, 13, 17). Therefore, folate inhibitors may not be of optimal efficacy for toxoplasmosis in pregnancy. The other drawback of using folate inhibitors (although sometimes counteracted by folinic acid supplementation and/or by combination with clindamycin, azithromycin, atovaquone, or cotrimoxazole) is that these inhibitors not only affect DNA synthesis in *T. gondii* tachyzoites but may also inhibit DNA synthesis in tissues with high metabolic activity, resulting in frequently reported bone marrow suppression (dose-related depression, more pronounced in immunocompromised patients) and dermatologic and gastrointestinal adverse effects (18, 19). For chemoprophylaxis in small ruminants (administration prior to infection), anticoccidial drugs have shown only limited protection (22% reduction of abortions) (20, 21). In the European Union, administration of antimicrobials in animals require a preceding laboratory diagnosis of the infection (Regulation 2019/6), such that decoquinate-medicated feed (Deccox) cannot be used for preventing *T. gondii* abortions.

Sheep models of congenital toxoplasmosis have emerged as a good alternative to mouse models for testing vaccines and drug candidates since (i) similar to congenital toxoplasmosis in humans (6), congenital *T. gondii* infection in sheep can result in abortion or stillbirth, while postnatal mortality of pups is the most common consequence of congenital infection in mice (22, 23); (ii) the mechanism of innate recognition of *T. gondii* infection through IFNγ-mediated immune responses of immunity-related GTPases (IRGs) that occurs in mice does not seem to have an equivalent in humans or sheep (24–26); and (iii) reproductive physiology-related features, such as the short period of gestation in mice and the large number of pups, make the results difficult to extrapolate to humans (27).

**Fig 1:**
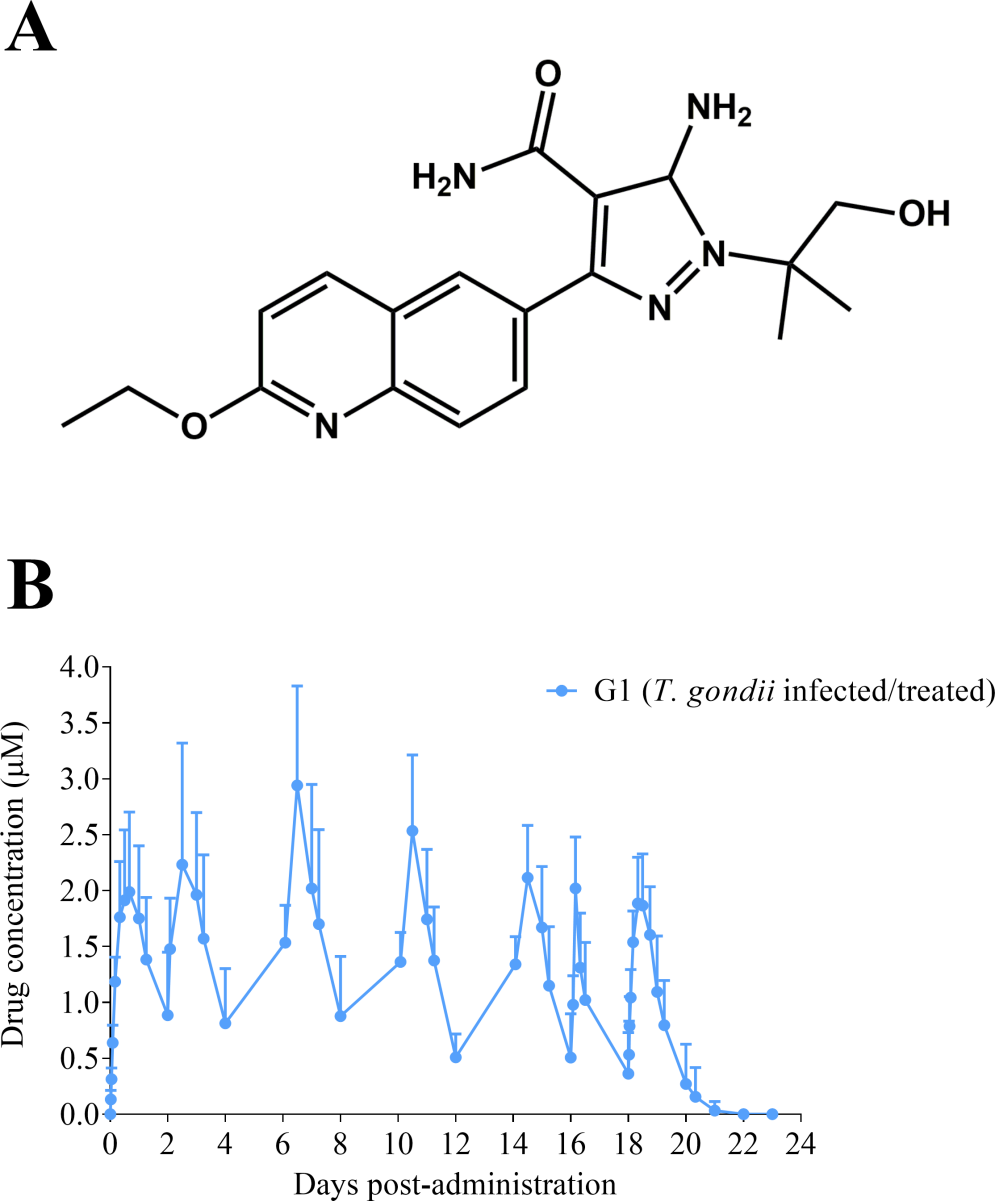
Chemical structure of BKI-1748 (**A**) and BKI-1748 plasma concentrations in the infected/treated dams (**B**). Treatment at 15 mg/kg was applied orally every 2 days up to 10 times. In B, mean concentrations + SD at the different sampling times are represented.

The last few decades have brought significant progress toward exploring parasite-targeted therapeutics and evaluation of existing drugs (28–31). Calcium-dependent protein kinase 1 (CDPK1) is very essential in *T. gondii* host cell invasion and egress and has no homologs in mammalian cells (32–37). TgCDPK1 can be selectively targeted by a class of ATP-competitive compounds, named bumped kinase inhibitors (BKIs) (38). Recent evidence suggests that some BKIs may also inhibit *T. gondii* by targeting TgMAPKL-1 (39, 40). BKI compounds from the 5-aminopyrazole-4-carboxamide (AC) scaffold, such as BKI-1748 (Fig. 1A), have shown an adequate safety window according to *in vitro* zebrafish embryo and pregnant mouse screening (41, 42). In addition, BKI-1748 has shown low interference with human ether-à-go-go-related gene (hERG) product, a potassium channel that plays an important role in regulating action potentials in myocardial cells, therefore exhibiting acceptable cardiovascular safety in dogs and rats (43, 44). However, affinity chromatography assessments identified some BKI-1748-binding proteins from zebrafish embryos involved in translation and RNA processing, suggesting potential off-target activity by BKI-1748 (45). Regarding efficacy, the effective concentration inhibiting *T. gondii* tachyzoite proliferation *in vitro* by 50% (EC_50_) of BKI-1748 was in the nanomolar range, and this compound was highly efficacious in non-pregnant and pregnant mouse models of toxoplasmosis (42, 43). In sheep, BKI-1748 did not show systemic or pregnancy-related toxicity and completely prevented reproductive failure and congenital infection when administered 2 days post-infection (p.i.) for 20 days (46).

Previous studies on efficacy of drug therapy for acute and congenital toxoplasmosis in mice (28, 29, 42, 47–49) and sheep (20, 21, 46, 50) started the treatment prior to or concomitantly to infection, or latest at 2 days after infection, when dissemination of *T. gondii* tachyzoites is unlikely to have been extensive, and the infection is still clinically undetected. In the present study, we sought to investigate the efficacy of BKI-1748 against congenital toxoplasmosis in sheep when the compound is administered from day 7 p.i. onward (at the time of fever peak and increased serum IFNγ levels), which may help to accurately estimate its efficacy in treating real-life acute congenital toxoplasmosis both in animals and humans. In addition, in this study, we attempted to mimic natural oocyst-driven infections in intermediate hosts, which are likely to be infected by a low dose of sporulated oocysts (51). Infection of pregnant sheep with a low dose of *T. gondii* oocysts causes late abortions and stillborn, lambs whereas a high dose of oocysts causes mainly early abortions (23).

## RESULTS

To summarize the experimental design, in group 1 (G1; infected/treated), 7 days after oral administration of 10 TgShSp1 oocysts to sheep at mid-pregnancy, BKI-1748 was orally applied 10 times at 15 mg/kg of body weight every 48 h. Sheep in group 2 (G2; infected/untreated) were infected with the same oocyst dose but did not receive the treatment. Sheep in group 3 (G3; uninfected/untreated) were used as sentinel control.

### Pharmacokinetics

BKI-1748 plasma concentrations in the dams from infected/treated group (G1) are shown in Fig. 1B. A mean (±standard deviation) AUC of 67.8 ± 11.5 h*µmol/L was observed for each BKI-1748 dose. A mean (±standard deviation) C_max_ of 2.29 ± 0.19 µM was reached in the plasma of the dams at 8–24 h (mainly at 12 h) after each BKI-1748 administration. Likewise, mean (±standard deviation) trough plasma concentrations of 0.6 ± 0.12 µM at 48 h after each BKI-1748 treatment. The average concentration (C_avg_) over the treatment course was 1.29 ± 0.8 µM. Total BKI-1748 plasma concentrations above the *in vitro* EC_50_ for *T. gondii* tachyzoites were maintained for 20 days (between days 7 to 27 p.i.). Considering that BKI-1748 shows plasma protein binding of 94.6% for sheep, the free drug concentration remains above the *in vitro* EC_50_ for 53% of the time during the 20-day treatment period.

### Rectal temperatures

**Fig 2:**
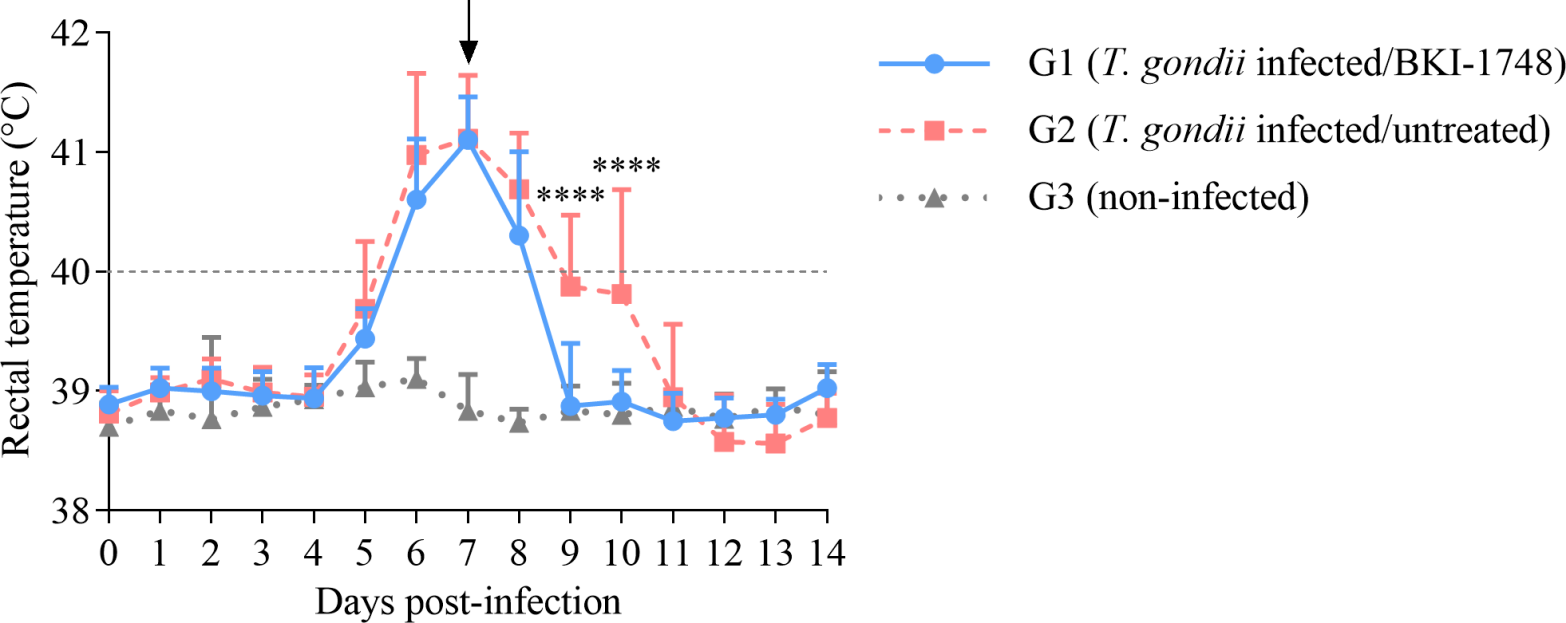
Rectal temperatures of *T. gondii*-infected and uninfected sheep, receiving or not receiving the treatment with BKI-1748. Each point represents the mean + SD for each group. Horizontal dashed line indicates the upper threshold for physiological rectal temperature in sheep, and the black arrow points the beginning of the treatment on day 7 p.i. For significant differences between infected groups, (****) indicates *P* < 0.0001.

Rectal temperatures increased significantly from day 5 (*P* < 0.05) to 10 (*P* < 0.001) p.i. in G2 (infected/untreated) and from day 6 to 8 p.i. (*P* < 0.0001) in G1 (infected/treated) compared with uninfected animals (G3). Comparing both infected groups, in the treated group (G1), rectal temperatures were significantly lower than in the untreated group (G2) on days 9 and 10 p.i. (*P* < 0.0001) (2 and 3 days after the treatment started) (Fig. 2).

### Cellular and humoral immune responses

**Fig 3:**
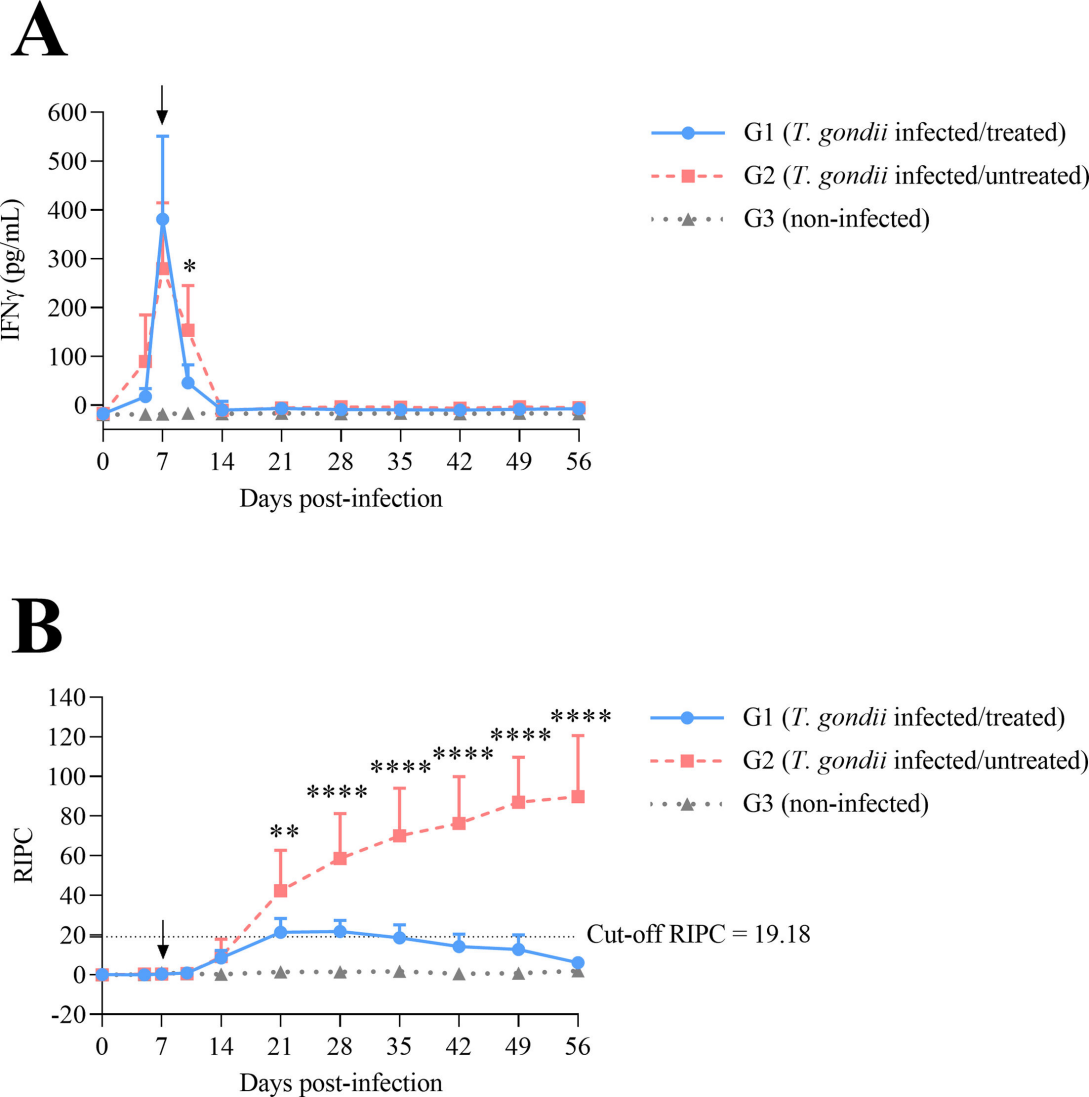
IFNγ levels (**A**) and anti-*T*. *gondii* IgG levels (**B**) in serum. In A, concentrations of IFNγ are expressed in pg/mL. In B, anti-*T*. *gondii* IgG levels are expressed in relative index percent (RIPC). Each point represents the mean + SD at the different sampling times for each group. The black arrow points the beginning of the treatment on day 7 p.i. Horizontal dashed lines in B indicate the cut-off (RIPC ≥19.18) for experimental conditions established by the previously validated TgSALUVET ELISA 2.0 (52). For significant differences between infected groups, (*) indicates *P* < 0.05, (**) indicates *P* < 0.01, and (****) indicates *P* < 0.0001.

IFNγ levels peaked in serum from the infected groups (G1 and G2) on day 7 p.i. with statistically significant differences on days 7 (*P* < 0.001) and 10 p.i. (*P* < 0.05) compared with uninfected animals (G3). From day 14 p.i. onward, IFNγ levels returned to baseline levels in the infected groups. Comparing both infected groups, animals from infected/treated group (G1) exhibited significantly lower IFNγ levels on day 10 p.i. (*P* < 0.05) compared with animals from infected/untreated group (G2) (Fig. 3A).

Examining the anti-*T*. *gondii* IgG serum levels in the dams (Fig. 3B), 7 out of 8 sheep from the infected/untreated group (G2) seroconverted on day 21 p.i., and the remaining sheep were seropositive from day 35 p.i. onward. In the infected/treated group (G1), 5 out of 8 sheep were seropositive on day 21 p.i., with relative index percent (RIPC) values of seropositive sheep on that day (mean ± standard deviation of 25.4 ± 5.2) close to cut-off value (RIPC ≥19.18), and all of them returned to seronegative by day 56 p.i. Comparing both infected groups, the infected and then treated sheep in G1 showed significantly lower IgG levels compared with sheep in G2 from day 21 p.i. (*P* < 0.01) until the final sampling on day 56 p.i. (*P* < 0.0001).

Concerning IgG titers of aborted fetuses and lambs, in the infected/untreated group (G2), aborted fetuses on day 13 p.i. were seronegative, whereas all stillborn and healthy lambs were seropositive (individual indirect fluorescent antibody test [IFAT] titers are shown in Table S2). In this group (G2), stillborn lambs showed higher IFAT titers (median IFAT titer of 1:1,600) than healthy lambs (median IFAT titer of 1:400), although differences were not statistically significant (*P* = 0.14). The aborted fetus as well as the lambs of the infected/treated group (G1) and the lambs of the non-infected group (G3) were seronegative.

### Pregnancy outcome

**Fig 4:**
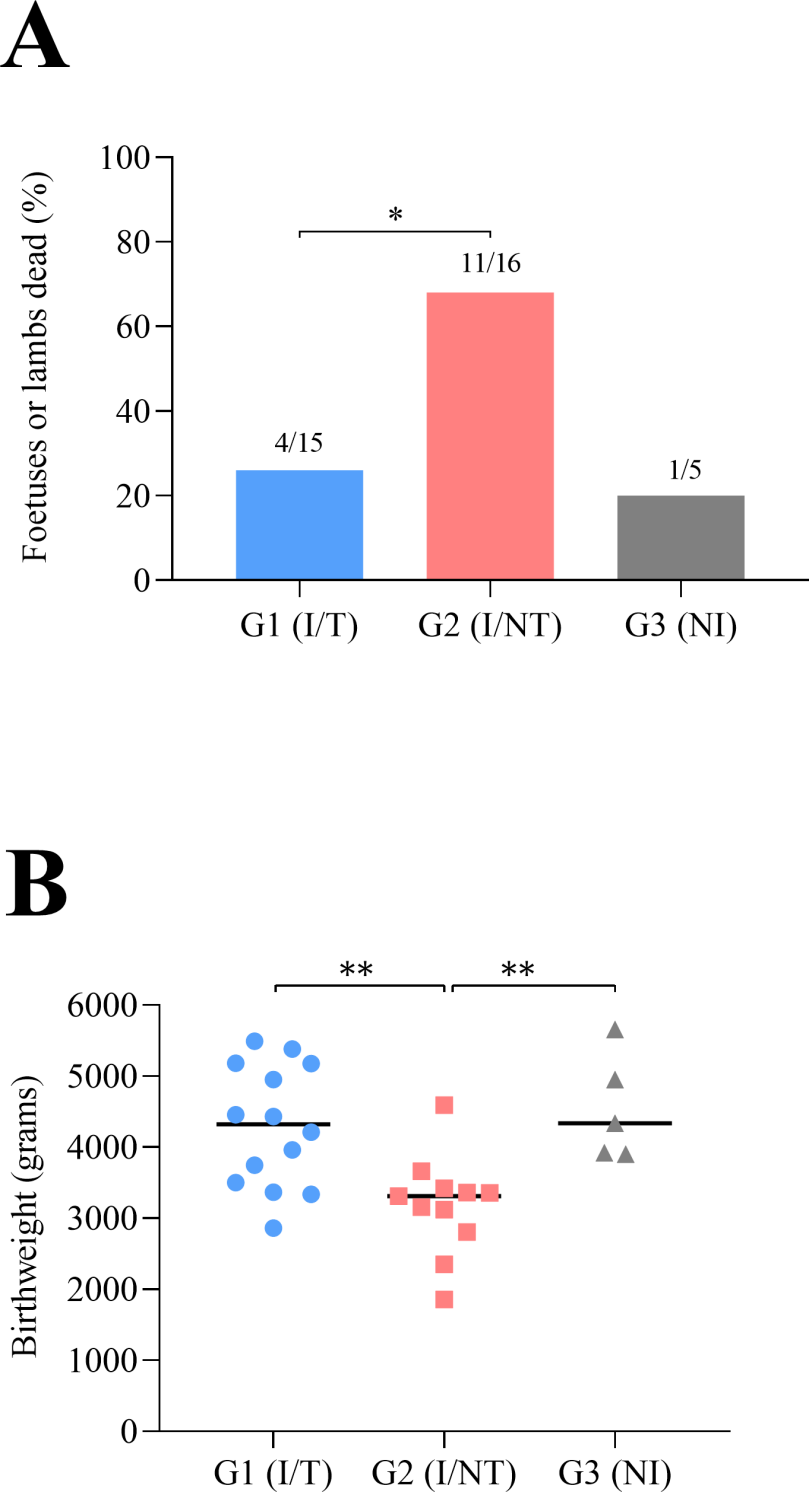
Lamb‘s viability (**A**) and birthweight of the lambs (**B**) born from *T. gondii*-infected (treated or not) and uninfected sheep. In A, dead fetuses/lambs include aborted/mummified fetuses and stillborn lambs (for more information about outcome for pregnancy see Table 1; Table S2). In B, the birthweights of lambs (including stillborn and healthy lambs) are shown. Birthweight was corrected by applying a correction factor depending on the number of lambs per sheep (see Material and Methods section). For significant differences, (*) indicates *P* < 0.05 and (**) indicates *P* < 0.01.

Fetal/lamb survival is detailed in Table 1 and Table S2. In both infected groups, 1 out of 8 sheep (12.5%) aborted (in the infected/untreated group on day 13 p.i. (early abortion) and in the infected/treated group on day 38 p.i.), and 7 out of 8 sheep (87.5%) gave birth, although one sheep from the infected/untreated group (G2) delivered prematurely on day 142 of pregnancy. In the infected/untreated group (G2), three mummified fetuses, six stillborn lambs, and five healthy lambs were observed at the time of delivery. By contrast, in the infected/treated group (G1) three stillborn lambs and 11 healthy lambs were observed. Thus, a significantly lower percentage of dead fetuses/lambs were found in G1 (infected/treated) (26.6%, 4/15) compared with G2 (infected/untreated) (68.7%, 11/16) (*P* < 0.05) (Fig. 4A). In the uninfected group (G3), only 1 out of 5 lambs (20%) were born dead, and no significant differences in lamb mortality were found when compared with G1, although when compared with G2, it was close to statistical significance (*P* = 0.12). Regarding birthweight of lambs born (including stillborn and healthy lambs), lambs from sheep in infected/untreated group (G2) exhibited significantly lower birthweights (23% reduction in the birthweight of the lambs) compared with G1 (infected/treated) and G3 (uninfected sheep) (*P* < 0.01), whereas no significant differences were found in lambs from sheep in G1 (infected/treated) compared with lambs born from the uninfected sheep (G3) (Fig. 4B). Furthermore, comparing birthweights of stillborn and healthy lambs of each group and between groups, lower birthweights of stillborn lambs in G2 (infected/untreated) were found when compared with stillborn lambs in G1 (infected/treated) (*P* < 0.05) (Table S2).

**TABLE 1:** Outcome for pregnancy and parasite DNA detection in target tissues from the dams and the offspring

Group	Dams			Offspring			
	Outcome for pregnancy^*a*^	n^*b*^	Parasite DNA detection in cotyledons (placenta)^*c*^	Clinical outcome	n^*d*^	Parasite DNA detection in the brain^*c*^	Parasite DNA detection in the lung^*c*^
Group 1 (infected, treated)	Abortion (38 d.p.i.)	1/8 (12.5%)	NA	Aborted fetuses	1/15 (6.7%)	0/3 (0%)	0/0 (0%)^*f*^
	Delivery (143–150 dg)	7/8 (87.5%)	0/42 (0%)	Mummified fetuses	0/15 (0%)	NA^*g*^	NA
				Stillborn lambs	3/15 (20%)	0/9 (0%)	0/9 (0%)
				Healthy lambs	11/15 (73.3%)	0/33 (0%)	0/33 (0%)
Group 2 (infected, untreated)	Abortion (13 d.p.i.)	1/8 (12.5%)	0/6 (0%)	Aborted fetuses	2/16 (12.5%)	0/6 (0%)	0/6 (0%)
	Delivery (142–149 dg)	7/8 (87.5%)^*e*^	40/40^*f*^ (100%)	Mummified fetuses	3/16 (18.7%)	6/6^*f*^ (100%)	4/4^*f*^ (100%)
				Stillborn lambs	6/16 (37.5%)	14/18 (77.8%)	18/18 (100%)
				Healthy lambs	5/16 (31.3%)	8/15 (53.3%)	15/15 (100%)
Group 3 (uninfected, untreated)	Abortion	0/3 (0%)	NA	Aborted fetuses	0/5 (0%)	NA	NA
	Delivery (146–150 dg)	3/3 (100%)	0/18 (0%)	Mummified fetuses	0/5 (0%)	NA	NA
				Stillborn lambs	1/5 (20%)	0/3 (0%)	0/3 (0%)
				Healthy lambs	4/5 (80%)	0/12 (0%)	0/12 (0%)

^*a*^ The numbers in the brackets indicate the day post-infection (d.p.i.) in which abortion/fetal mortality was detected or day of gestation (dg) in which delivery occurred.

^*b*^ Number of dams/total number of dams; percentage in brackets.

^*c*^ Number of PCR-positive samples/total number of samples analyzed, percentage in brackets. Six different cotyledons from each sheep and three replicates of brain and lung from each fetus/lamb were submitted to PCR.

^*d*^ Number of fetuses/lambs in each category of clinical outcome for the offspring/Total number of fetuses/lambs; percentage in brackets

^*e*^ One sheep had a premature delivery on day 142 of pregnancy.

^*f*^ Degraded DNA in some samples (see Supplementary file 2).

^*g*^ NA: not available samples for parasite DNA detection due to placentophagy (cotyledons from the aborted sheep in G1) or due to the lack of dams or fetuses/lambs in the corresponding category.

### Parasite detection in placental and fetal/lamb tissues

Parasite DNA detection in target placental and fetal/lamb tissues is summarized in Table 1 and detailed in Table S2. In the infected/untreated group (G2), parasite DNA was not detected in the cotyledons and fetal tissues from the aborted fetus on day 13 p.i. However, in G2 sheep that delivered lambs, parasite DNA was found in all samples from placentas (cotyledons) and the lungs of the lambs. In brain tissues of all but one of the G2 lambs, there was at least one out of three positive samples, and a higher parasite DNA detection rate was found in stillborn lambs (77.8%) compared with healthy lambs (53.3%), although differences were not statistically significant (*P* = 0.16).

In the infected/treated group (G1), parasite DNA was not detected in any of the samples from the placenta (cotyledons), aborted fetus, or lambs born. The lack of parasite DNA was also confirmed in samples from the uninfected group (G3).

## DISCUSSION

Congenital toxoplasmosis is a well-known cause of fetal loss, neurological, and ocular disorders in humans (5) and is also relevant in small ruminants, decreasing the number of live lambs (53). In humans, successful outcomes of congenital toxoplasmosis therapy using folate inhibitors, such as pyrimethamine and sulfadiazine is influenced by how promptly the treatment is initiated after *T. gondii* infection of the pregnant mother (window of opportunity if started at 3 weeks of infection) (4). Adverse events derived from the treatment with folate inhibitors, requiring treatment discontinuation and/or change in therapy, involved up to 37% of treated patients (13, 14, 16–19, 31). Furthermore, pyrimethamine is teratogenic (major malformations, such as neural tube defects), and its use in the first trimester is contraindicated (54). Therefore, there is an urgent need for the evaluation of the *in vivo* efficacy of well-tolerated therapeutic compounds.

BKI-1748 is an excellent candidate since administration from day 2 p.i. onward provided full protection against abortion and congenital *T. gondii* infection in sheep (46). However, the efficacy of BKI-1748 administered at later timepoints after infection has not been assessed so far. This study evaluated the efficacy of BKI-1748 applied from day 7 p.i. (and continued for 20 days) at an early stage of systemic parasite dissemination (when rectal temperature and IFNγ reached maximum levels) but before *T. gondii* invasion of sheep placenta took place (23, 55)

The BKI-1748 drug regimen had been shown to be safe in pregnant sheep (46), and therefore, as expected, no systemic or pregnancy-related signs of toxicity were observed throughout the study. BKIs exhibit an *in vitro* parasitostatic effect (56), and therefore, we studied the previously described extended treatment of BKI-1748 with 10 doses at 15 mg/kg every 2 days (46), obtaining similar total plasma drug levels. Pregnant sheep reached maximum plasma concentrations of 2–3 µM and maintained for 20 days at total plasma concentrations above the EC_50_ for *T. gondii* (0.063 µM)(42, 43)*,* although considering plasma–protein binding of BKI-1748 to sheep plasma, free BKI-1748 falls below the EC50 for approximately half of the dosing period. It is not clear whether BKIs are time-dependent or concentration-dependent anti-parasitics, so we are uncertain if they need to be maximized for the time that free (unbound) drug concentrations spend above the *in vitro T. gondii* EC50 (time-dependent) or maximized for the free peak-to-EC50 ratio (concentration-dependent) (57). Given the success of this regimen, we suspect BKIs are time-dependent for anti-*T*. *gondii* therapy.

Type II *T. gondii* lineage is predominant in Europe and North America (58, 59); therefore, the recently *in vitro* and *in vivo* characterized Type II isolate, TgShSp1, was used for testing drug efficacy (23, 55, 60–62). Sheep experimentally infected with *T. gondii* oocysts usually develop fever for some days during the first 2 weeks post-infection (53). After infection with 10 TgShSp1 oocysts, fever was found in infected/untreated pregnant sheep between days 5 and 10 post-infection, consistent with previous results (23, 55). A decrease in rectal temperature was observed at 2–3 days after beginning BKI-1748 administration (9–10 days after infection), which very likely indicates an early reduction of parasite replication as previously described in studies evaluating BKIs in sheep (46, 50). Regarding cellular immune response, IFNγ is a pivotal cytokine of host immune response to *T. gondii* infection that controls parasite replication, although the mechanism varies between hosts (26). Similar to the present study in which an increase of serum IFNγ was detected between days 7 and 10 post-infection in the infected/untreated group, a previous study reported an increase of serum IFNγ levels between days 5 and 8 post-infection following an infection with 50 oocysts of the *T. gondii* M4 isolate (63). Administration of BKI-1748 resulted in lower IFNγ levels on day 10 p.i., probably due to the control of parasite replication, which has been previously found in studies evaluating anti-*T*. *gondii* drugs (64–67). For humoral immune responses, infected but untreated sheep seroconverted around 3 weeks after infection, as it has been previously described (23, 52). In contrast, infected/treated sheep had low anti-*T*. *gondii* IgG levels (below or slightly above the cut-off value), which probably indicates that antigen stimulation in the treated group was very low throughout the study. While early BKI-1748 treatment starting 2 days after infection did not lead to seroconversion (46), an equivalent regimen starting 7 days after infection resulted in some sheep that were slightly seropositive at several time points. However, the IgG levels remained low, and all infected/treated animals at the end of the study were seronegative, supporting the fact that there is no parasite reactivation from BKI-1748 clearance (117 days of pregnancy, i.e., 27 days after infection) to delivery time (150 days of pregnancy) as it has been previously described (46). These results contrast with a previous study with the pyrazolopyrimidine, BKI-1294, where half of the animals seroconverted by the end of therapy (50).

Fetal and lamb mortality and morbidity have been shown to be dependent on the dose of *T. gondii* oocysts that infect sheep during pregnancy (23). An infectious dose of 10 TgShSp1 oocysts administered at mid-pregnancy was previously reported to result in 0%–20% early abortions on days 11–12 post-infection, and 16% experiencing late abortion/premature delivery (around 140 days of pregnancy) and a large proportion (53%) of stillborn lambs (23, 55), which is consistent with the results of this study for the infected/untreated group. The presence of some mummified fetuses at delivery (but detected to be dead before, around 22–26 days post-infection) described here was previously reported in experimental infections using a low dose of oocysts in pregnant sheep (55, 68) and under natural conditions (69, 70). The TgShSp1 isolate has a high tropism for placental and fetal tissues in sheep (55); tachyzoite replication in such tissues could weaken the lambs and stunt their growth, which may explain the high percentage of stillborn lambs found, although there are many additional factors affecting lamb mortality/morbidity, such as the number of lambs per sheep (71). The presence of stillborn lambs after *T. gondii* infection has been described under natural conditions (70, 72) and upon experimental infections at mid-pregnancy (73, 74). Additionally, the congenital *T. gondii* infection also triggers a reduction in the birthweight of the lambs, as it has been already recorded in sheep (20, 75) and humans (4). One of the limitations of our work in assessing viability in healthy lambs is that it was only assessed up to 48 h post-delivery, while in humans, the clinical signs of *T. gondii* infection in neonates may not appear until several years after birth (5, 6). Treatment with BKI-1748 significantly reduced fetal and lamb mortality and morbidity. Nevertheless, in the infected/treated group, one sheep aborted on day 38 post-infection, although the cause of abortion could not be determined since the dam was negative for anti*-T*. *gondii* antibodies as well as seronegative for other infectious agents causing abortion (see Material and Methods section), and *T. gondii* DNA was not detected in the fetal brain. In the infected/treated group, 20% of stillborn lambs were found, but the death of these lambs could not be associated with *T. gondii* infection as they were PCR negative (although the possibility of parasite-associated damage with a posterior parasite elimination cannot be ruled out), the same percentage of stillborn lambs was observed in the uninfected group, and they were heavier at birth than stillborn lambs in the infected/untreated group.

Sheep-to-lamb transmission of infection was 100% for the untreated/infected group with 10 TgShSp1 oocysts at 90 days of gestation, except for 2 out of 16 *T*. *gondii*-free fetuses that suffered early abortion on day 13 p.i., as previously described for parasite detection percentages and antibody titers in lambs (23). It is of interest to note that in the infected/untreated group from this study, stillborn lambs exhibited higher antibody titers and higher detection percentage in the brain than those born healthy, although differences were not statistically significant. By contrast, lambs born from infected dams treated from day 7 post-infection were seronegative for *T. gondii* antibodies and PCR negative in tissues (both fetal tissues and placental cotyledons), as reported for an earlier treatment from day 2 post-infection (46).

In conclusion, treatment with BKI-1748 was highly effective against fetal and lamb mortality/morbidity and vertical transmission in sheep infected at mid-pregnancy when applied from day 7 after *T. gondii* infection, which provided BKI-1748 exposure for 20 days. Additional studies will be needed to define the minimal therapeutic exposure required for a successful therapy and to evaluate efficacy with further postponement of treatment initiation, around 12–14 days after infection, when parasite invasion of sheep placenta is known to commence (55).

## MATERIALS AND METHODS

### Experimental design

The experimental design is summarized in Table 2. Nineteen pure Rasa Aragonesa breed pregnant sheep aged 18 months were selected from a commercial flock. All animals were seronegative for *T. gondii*, *N. caninum*, Border disease virus (BDV), Schmallenberg virus (SBV), *Coxiella burnetii*, and *Chlamydia abortus* as determined by enzyme-linked immunosorbent assay (ELISA). At mid-pregnancy, 90 days of gestation (dg), sheep from group 1 (G1) and group 2 (G2) were orally challenged with 10 T. *gondii* sporulated oocysts (stored at 4°C during 13 months after sporulation) of the *T. gondii* isolate TgShSp1 (PCR-RFLP genotype 3) (23). Seven days later, sheep from G1 were treated orally with 10 doses of BKI-1748 (Fig. 1A) at 15 mg/kg every 48 h, following the dosing protocol previously described (46). Compound was dissolved at 30 mg/mL in a vehicle containing 60% PHOSAL 53 medium-chain triglyceride (MCT) emulsion, 30% PEG400, and 10% ethanol.

**TABLE 2:** Experimental design

Group	Number of pregnant sheep	Number of fetuses/lambs	Challenge (P.O.)^*a*^	Treatment (P.O.)
G1	8	15	10 TgShSp1 sporulated oocysts	BKI-1748, 10 doses at 15 mg/kg q.o.d.^*b*^, starting at 7 days post-infection
G2	8	16	10 TgShSp1 sporulated oocysts	None
G3	3	5	PBS	60% Phosal 53 MCT, 30% PEG400, 10% Ethanol 96°C (vehicle), 10 doses q.o.d.

^*a*^ P.O.: *per os*, orally.

^*b*^ q.o.d.: every other day.

Rectal temperatures were recorded from 0 to 14 days p.i. daily and weekly afterward. For determination of BKI-1748 plasma concentrations, blood samples were collected from G1 into 1 mL tubes containing lithium heparin (Aquisel, Barcelona, Spain) following the time schedule previously described (46). Heparinized blood samples were centrifuged at 805*×g* for 30 min at 4°C, and plasma samples were stored at −20°C until analysis by liquid chromatography–tandem mass spectrometry (LC-MS/MS). For evaluation of peripheral immune responses, blood samples were collected on days 0, 5, 7, and 10 p.i. and then weekly until 56 days p.i. (just before delivery) in 4 mL vacutainer tubes without anticoagulant (Becton Dickinson, New Jersey, United States). After clotting, serum samples were stored at −80°C until analysis.

Transabdominal ultrasound scanning was performed weekly to evaluate fetal movements, heart beats, and the presence of hyperechoic amniotic fluid. Deliveries at 142 dg or before were considered premature deliveries (76). Premature and at term lambs were weighed just after birth. Birthweight of the lambs is influenced by the type of gestation (single, twin, etc.) (71); therefore, in order to accurately compare the birthweights of the lambs born in this study, correction factors were calculated using the birthweights of lambs born from the same breed and mating batch (Table S1). According to their survival, fetuses/lambs were classified into four categories: (i) aborted fetuses suffering early abortions (i.e., until day 14 p.i.) or late abortions (i.e., from 15 days p.i. to 51 days p.i., which corresponds to 141 dg); (ii) mummified fetuses (i.e., dead in the post infection stage but maintained *in utero* until delivery); (iii) stillborn lambs (i.e., born dead or dead during the first 2 days after birth); and (iv) healthy lambs (i.e., lambs born alive and without clinical signs during the first 2 days after birth). In lambs born alive, precolostral serum was collected immediately after delivery. To prevent any transmission of colostral antibodies from dams, udders were covered with a piece of cloth 1 week before the expected date of delivery as a preventive measure for night deliveries. Serum samples were stored at −80°C until analysis. Two days after delivery, healthy lambs and dams were sedated with xylazine (Rompun, Elanco, Monheim, Germany) and then euthanized by an intravenous overdose of embutramide and mebezonium iodide (T61, Intervet, Salamanca, Spain).

During necropsy, six randomly selected cotyledons were recovered from each placenta and stored at −80°C for further DNA extraction and PCR analysis. Samples from brain and lungs from aborted fetuses, mummified fetuses, and lambs (stillborn and healthy lambs) were stored at −80°C for DNA extraction and PCR analysis. Thoracic fluid was also collected from aborted fetuses and lambs born dead and maintained at −80°C for serology.

### Study of drug pharmacokinetics, immune responses, and parasite DNA detection

BKI-1748 plasma concentrations were determined as previously described (43). Calculations of maximum concentration (C_max_) for each dose, and area-under-the-curve (AUC) were determined using GraphPad Prism 8.0.1 software (San Diego, CA, USA).

For immune responses, IFNγ levels in serum were evaluated using a commercial bovine enzyme immunoassay that shows cross-reactivity with ovine IFNγ (3119–1H-6, Mabtech AB, Nacka, Sweden). *T. gondii*-specific IgG levels in dams’ sera were determined by a previously validated in house TgSALUVET ELISA 2.0 (cut-off for experimental conditions, RIPC ≥19.18) (52). In thoracic fluid and precolostral sera collected from aborted fetuses/dead lambs or lambs born alive, respectively, *T. gondii*-specific IgG levels were evaluated by indirect fluorescent antibody test (IFAT) (77), using an anti-sheep IgG (F5137, Sigma-Aldrich, Madrid, Spain) diluted 1:200 in Evans Blue. Fetal fluids and precolostral sera were diluted at twofold serial dilutions in PBS starting at 1:8 (for fetal fluids) and 1:50 (for precolostral sera) up to the endpoint titer. Continuous tachyzoite membrane fluorescence at a dilution of ≥1:8 for fetal fluids or ≥1:50 for precolostral sera was considered a positive reaction. Genomic DNA extraction from 50–100 mg of (i) six samples of different cotyledons (placenta) per dam, (ii) three samples for each lamb´s brain, and (iii) three samples of each lamb’s lungs was carried out using the commercial Maxwell RSC Tissue DNA Kit (Promega, Wisconsin, USA) as previously described (50). *T. gondii* DNA detection was carried out by an ITS1 PCR adapted to a single tube following procedures previously described (78).

### Statistical analysis

Rectal temperatures and cellular and humoral immune responses in the dams were analyzed using two-way ANOVA of repeated measures test. The number of dead fetuses/lambs (aborted fetuses, mummified fetuses, and stillborn lambs) and healthy lambs was compared between the different groups using the Fisher’s exact F-test. Corrected birthweights of the lambs were compared using the non-parametric Kruskal–Wallis test followed by Dunn’s test for comparisons between groups, as well as the Mann–Whitney test for pairwise comparisons. In G2 (infected/untreated), stillborn lambs and healthy lambs were compared for IFAT titers in precolostral sera using Mann–Whitney test and for differences in frequency of parasite DNA detection in the brain using Fisher’s exact F-test. Statistical significance for all analyses was established at *P* < 0.05. All statistical analyses were performed using GraphPad Prism 8.0.1 software (San Diego, CA, USA).

## SUPPLEMENTAL MATERIAL

Table S1 aac.01448-24-s0001.docx Birth weights of uninfected lambs from Rasa Aragonesa sheep.

Table S2 aac.01448-24-s0002.docx Individual data of clinical outcome and infection status of the offspring.

ASM does not own the copyrights to Supplemental Material that may be linked to, or accessed through, an article. The authors have granted ASM a non-exclusive, world-wide license to publish the Supplemental Material files. Please contact the corresponding author directly for reuse.
