# Supplementary material for: Treatment with BKI-1748 after Toxoplasma gondii systemic dissemination in experimentally infected pregnant sheep improves fetal and lamb mortality and morbidity and prevents congenital infection
Source: Antimicrob Agents Chemother. 2024 Dec 31;69(2):e01448-24. doi: 10.1128/aac.01448-24 (PMC11823607; doi:10.1128/aac.01448-24)
Supplement: Table S1 — Birth weights of uninfected lambs from Rasa Aragonesa sheep. [file aac.01448-24-s0001.docx]

|  | **Single pregnancies (g)** | **Twin pregnancies (g)** | | **Triplet pregnancies (g)** | **Quadruplet pregnancies (g)** |
| --- | --- | --- | --- | --- | --- |
|  | 4439 | 4315 | 3500 | 3365 | 1720 |
|  | 4585 | 3772 | 3100 | 3060 | 1756 |
|  | 5000 | 3950 | 3430 | 3260 | 2423 |
|  | 4310 | 3711 | 3400 | 2200 | 2227 |
|  | 4225 | 3385 | 4590 | 1530 | 1800 |
|  | 2632 | 4350 | 3925 | 2100 | 2060 |
|  | 5580 | 3800 | 3580 | 3100 | 1600 |
|  | 4400 | 4100 | 3500 | 2900 | 1600 |
|  | 5100 | 3085 | 4200 | 1720 | 1700 |
|  | 4484 | 2950 | 3900 | 1720 | 2750 |
|  | 3930 | 4234 | 3000 | 1720 | 3100 |
|  | 4280 | 4014 | 3400 | 2750 | 1850 |
|  | 5100 | 4340 | 3200 | 2480 |  |
|  | 5800 | 3260 | 4200 | 2300 |  |
|  | 4100 | 3080 | 3600 | 2240 |  |
|  | 4400 | 2680 | 3600 | 2200 |  |
|  | 3600 | 3958 | 3518 | 2500 |  |
|  | 5400 | 3150 | 2820 | 2200 |  |
|  | 3900 | 4150 | 2905 |  |  |
|  | 4850 | 3120 | 4050 |  |  |
|  | 4350 | 4000 | 3500 |  |  |
|  | 4820 | 4750 | 3732 |  |  |
|  | 4900 | 3250 | 3640 |  |  |
|  | 5350 | 3800 | 3500 |  |  |
|  | 3200 |  |  |  |  |
|  | 3350 |  |  |  |  |
|  | 3300 |  |  |  |  |
|  | 4440 |  |  |  |  |
|  | 3550 |  |  |  |  |
|  | 4305 |  |  |  |  |
|  | 4050 |  |  |  |  |
|  | 3240 |  |  |  |  |
|  |  |  |  |  |  |
| Mean (g) | 4343 | 3646 | | 2408 | 2049 |
| **Correction factor** | **1** | **1,19** | | **1,80** | **2,11** |
|  |  |  |  |  |  |

**Table S1. Birthweights of uninfected lambs from Rasa Aragonesa sheep.**
