# Supplementary material for: Treatment with BKI-1748 after Toxoplasma gondii systemic dissemination in experimentally infected pregnant sheep improves fetal and lamb mortality and morbidity and prevents congenital infection
Source: Antimicrob Agents Chemother. 2024 Dec 31;69(2):e01448-24. doi: 10.1128/aac.01448-24 (PMC11823607; doi:10.1128/aac.01448-24)
Supplement: Table S2 — Individual data of clinical outcome and infection status of the offspring. [file aac.01448-24-s0002.docx]

Table S2. Individual data of clinical outcome and infection status of the offspring

| **Group** | **Sheep ref.** | **Outcome for pregnancy^a^** | **Foetus/lamb ref.** | **Clinical outcome for the offspring** | **Birthweight (g)** | **Corrected birthweight^b^ (g)** | **IFAT^c^** | **Parasite DNA detection^d^** | |
| --- | --- | --- | --- | --- | --- | --- | --- | --- | --- |
|  |  |  |  |  |  |  |  | **Lambs´s brain** | **Lambs´s lung** |
| Group 1 | 1.1 | Delivery (150 dg) | 1.1 F1 | Healthy lamb | 4430 | 4430 | - | - | - |
|  | 1.2 | De1ivery (149 dg) | 1.2 F1 | Healthy lamb | 2805 | 3338 | - | - | - |
|  |  |  | 1.2 F2 | Healthy lamb | 2403 | 2860 | - | - | - |
|  | 1.3 | Delivery (149 dg) | 1.3 F1 | Healthy lamb | 3500 | 3500 | - | - | - |
|  | 1.4 | Delivery (146 dg) | 1.4 F1 | Healthy lamb | 2990 | 5382 | - | - | - |
|  |  |  | 1.4 F2 | Healthy lamb | 3050 | 5490 | - | - | - |
|  |  |  | 1.4 F3 | Stillborn lamb | 2200 | 3960 | NA | - | - |
|  | 1.5 | Delivery (150 dg) | 1.5 F1 | Healthy lamb | 4950 | 4950 | - | - | - |
|  | 1.6 | Delivery (148 dg) | 1.6 F1 | Healthy lamb | 2476 | 4457 | - | - | - |
|  |  |  | 1.6 F2 | Stillborn lamb | 2082 | 3747 | - | - | - |
|  |  |  | 1.6 F3 | Stillborn lamb | 2877 | 5179 | - | - | - |
|  | 1.7 | Abortion (38 dpi) | 1.7 F1 | Aborted foetus | NA | NA | - | - | * |
|  | 1.8 | Delivery (143 dg) | 1.8 F1 | Healthy lamb | 1871 | 3368 | - | - | - |
|  |  |  | 1.8 F2 | Healthy lamb | 2340 | 4212 | - | - | - |
|  |  |  | 1.8 F3 | Healthy lamb | 2880 | 5184 | - | - | - |
| Group 2 | 2.1 | Delivery (147 dg) | 2.1F1 | Healthy lamb | 3360 | 3360 | 1:6400 | +++ | +++ |
|  | 2.2 | Delivery (149 dg) | 2.2 F1 | Healthy lamb | 2652 | 3155 | 1:400 | + | +++ |
|  |  |  | 2.2 F2 | Healthy lamb | 2358 | 2806 | 1:100 | - | +++ |
|  | 2.3 | Delivery (145 dg) | 2.3 F1 | Healthy lamb | 4594 | 4594 | 1:400 | + | +++ |
|  | 2.4 | Abortion (13 dpi) | 2.4 F1 | Aborted foetus | NA | NA | - | - | - |
|  |  |  | 2.4 F2 | Aborted foetus | NA | NA | - | - | - |
|  | 2.5 | Delivery (143 dg) | 2.5 F1 | Mummified foetus | NA | NA | NA | +++ | +++ |
|  |  |  | 2.5 F2 | Stillborn lamb | 1621 | 3420 | 1:400 | +++ | +++ |
|  |  |  | 2.5 F3 | Healthy lamb | 1569 | 3311 | 1:200 | +++ | +++ |
|  |  |  | 2.5 F4 | Mummified foetus | NA | NA | NA | +++ | +* |
|  | 2.6 | Delivery (145 dg) | 2.6 F1 | Stillborn lamb | 1858 | 1858 | 1:800 | + | +++ |
|  | 2.7 | Delivery (147 dg) | 2.7 F1 | Stillborn lamb | 2819 | 3355 | 1:1600 | + | +++ |
|  |  |  | 2.7 F2 | Mummified foetus^#^ | NA | NA | NA | * | * |
|  | 2.8 | Delivery (142 dg) ^α^ | 2.8 F1 | Stillborn lamb | 1734 | 3121 | 1:1600 | +++ | +++ |
|  |  |  | 2.8 F2 | Stillborn lamb | 2035 | 3663 | 1:6400 | +++ | +++ |
|  |  |  | 2.8 F3 | Stillborn lamb | 1308 | 2354 | 1:3200 | +++ | +++ |
| Group 3 | 3.1 | Delivery (150 dg) | 3.1 F1 | Healthy lamb | 3900 | 3900 | - | - | - |
|  | 3.2 | Delivery (146 dg) | 3.2 F1 | Healthy lamb | 2180 | 3924 | - | - | - |
|  |  |  | 3.2 F2 | Healthy lamb | 2410 | 4338 | - | - | - |
|  |  |  | 3.2 F3 | Stillborn lamb | 3144 | 5659 | - | - | - |
|  | 3.3 | Delivery (150 dg) | 3.3 F1 | Healthy lamb | 4950 | 4950 | - | - | - |

^a^ Day post-infection (dpi) in which abortion/fetal mortality was detected or day of gestation (dg) in which delivery occurred. (^α^) Premature delivery on day 142 of pregnancy. ^#^ Found dead by ultrasound scanning on day 23 post infection and mummified at delivery (57 days post infection).

^b^ Birthweight after applying the correction factor for twin (x1.25) triplet (x1.75) and quadruplet (x 2.23) pregnancies (see Supplementary file 2).

^c^ IFAT IgG antibody titers in foetal body fluids from aborted foetuses and in precolostral serum from lambs.

^d^ Parasite DNA detection in none (-), <33% (+), 33-66% (++) and >66% (+++) of the analyzed samples. (*) means degraded DNA.

NA: not available
